# Osteosarcopenia increases the risk of mortality: a systematic review and meta-analysis of prospective observational studies

**DOI:** 10.1007/s40520-024-02785-9

**Published:** 2024-06-18

**Authors:** Nicola Veronese, Francesco Saverio Ragusa, Shaun Sabico, Ligia J. Dominguez, Mario Barbagallo, Gustavo Duque, Nasser Al-Daghri

**Affiliations:** 1 Geriatric Unit, Department of Internal Medicine and Geriatrics, University of Palermo, Palermo, 90127 Italy; 2 Chair for Biomarkers of Chronic Diseases, Biochemistry Department, College of Science, King Saud University, Riyadh, 11451 Saudi Arabia; 3 Department of Medicine and Surgery, Kore University of Enna, Enna, 94100 Italy; 4 Bone, Muscle & Geroscience Group, Research Institute of the McGill University Health Centre, Montreal, QC Canada; 5 Dr Joseph Kaufmann Chair in Geriatric Medicine, Department of Medicine, McGill University, Montreal, QC Canada

**Keywords:** Osteosarcopenia, Meta-analysis, Mortality, Osteoporosis, Sarcopenia

## Abstract

**Background & aims:**

Osteosarcopenia is a recently recognized geriatric syndrome. The association between osteosarcopenia and mortality risk is still largely underexplored. In this systematic review with meta-analysis of prospective cohort studies, we aimed to explore whether osteosarcopenia could be associated with a higher mortality risk.

**Methods:**

Several databases were searched from the inception to 16th February 2024 for prospective cohort studies dealing with osteosarcopenia and mortality. We calculated the mortality risk in osteosarcopenia vs. controls using the most adjusted estimate available and summarized the data as risk ratios (RRs) with their 95% confidence intervals (CIs). A random-effect model was considered for all analyses.

**Results:**

Among 231 studies initially considered, nine articles were included after exclusions for a total of 14,429 participants (mean age: 70 years; 64.5% females). The weighted prevalence of osteosarcopenia was 12.72%. Over a mean follow-up of 6.6 years and after adjusting for a mean of four covariates, osteosarcopenia was associated with approximately 53% increased risk of mortality (RR: 1.53; 95% CI: 1.28–1.78). After accounting for publication bias, the re-calculated RR was 1.48 (95%CI: 1.23–1.72). The quality of the studies was generally good, as determined by the Newcastle Ottawa Scale.

**Conclusions:**

Osteosarcopenia was significantly linked with an increased risk of mortality in older people, indicating the need to consider the presence of osteoporosis in patients with sarcopenia, and vice versa, since the combination of these two conditions typical of older people may lead to further complications, such as mortality.

**Supplementary Information:**

The online version contains supplementary material available at 10.1007/s40520-024-02785-9.

## Introduction

Osteosarcopenia is a term derived from “osteo” (bone) and “sarcopenia” (loss of muscle mass and strength) [1]. This condition refers to the concurrent presence of osteoporosis and sarcopenia, two age-related musculoskeletal conditions with significant implications for health and functional independence in older adults [1]. While osteoporosis and sarcopenia have traditionally been viewed as distinct entities, emerging evidence suggests that they often coexist and share common pathophysiological mechanisms, leading to a synergistic decline in musculoskeletal health [2]. 

Nowadays, the importance of osteosarcopenia lies in its profound impact on overall health, mobility, and quality of life in older individuals [3]. On the one hand, osteoporosis, characterized by low bone mass and microarchitectural deterioration of bone tissue, increases the risk of fragility fractures, particularly in the spine, hip, and wrist, resulting in pain, disability, and loss of independence [4]. Sarcopenia, on the other hand, involves the progressive loss of muscle mass, strength, and function, leading to impaired physical performance, increased risk of falls, and functional decline [5]. 

Probably, the coexistence of osteoporosis and sarcopenia in osteosarcopenia further exacerbates these adverse outcomes, creating a vicious cycle of frailty, disability, and mortality in older adults [6]. Individuals with osteosarcopenia are at heightened risk of falls, fractures, hospitalizations, and institutionalization, placing a substantial burden on healthcare systems and society as a whole [7]. 

Understanding the etiology, epidemiology, and clinical consequences of osteosarcopenia is essential for developing effective prevention and management strategies to optimize musculoskeletal health and promote healthy aging. In this regard, the association between osteosarcopenia and mortality is still underexplored.

Given this background, with this systematic review and meta-analysis of prospective cohort studies, we aimed to explore whether osteosarcopenia could be associated with a higher mortality risk.

## Methods

This systematic review and meta-analysis was conducted in accordance with the updated 2020 Preferred Reporting Items for Systematic Reviews and Meta-Analyses (PRISMA) guidelines [8]. The protocol has been registered in Open Science Framework (https://osf.io/5drnu/).

### Search strategy

Two independent reviewers (NV and FSR) searched PubMed, Web of Science, and Embase from inception until 16 February 2024. The full search strategy and the search terms used are described in **Supplementary Table 1**. Discrepancies in the literature search process were resolved by a third investigator (SS).

### Inclusion and exclusion criteria

Studies were included based on the following criteria: (i) Baseline data from observational prospective studies; (ii) clear diagnostic criteria for osteosarcopenia indicated as validated criteria for osteoporosis and for sarcopenia; (iii) reporting data regarding mortality and summarizing these data as hazard ratios (HRs) or risk ratios (RRs), deriving from multivariate analyses; and (iv) studies had to include both adults with and without osteosarcopenia. Published articles were excluded if they (i) were reviews, letters, in vivo or in vitro experiments, commentaries, or posters; and (ii) were not published as a full text and in English, since literature has demonstrated excluding such papers has little impact on the effect estimates and conclusions of systematic reviews [9].

### Data extraction and risk of bias

Two authors (NV and FSR) extracted data independently, which included name of first author, date of publication, country of origin, participant age, study design, population studied, number of participants, definition of sarcopenia and osteoporosis, tools and criteria for assessing sarcopenia and osteoporosis, follow-up time in years, main condition, number and type of adjustments in statistical analyses. Disagreements between reviewers were resolved by one independent reviewer (SS).

The Newcastle-Ottawa Scale (NOS) was used to assess the study quality/risk of bias [10]. The NOS assigns a maximum of 9 points based on three quality parameters: selection, comparability, and outcome. The evaluation was made by two investigator (FSR and NV) and checked by another (SS). The risk of bias was consequently categorized as high (< 5/9 points), moderate (6–7), or low (8–9) [11].

### Outcomes

The outcome of our interest was mortality (overall or specific), reported using any method, including death certificates, medical records, administrative data, or other information, such as asking for information from relatives.

### Statistical analysis

The primary analysis compared the cumulative incidence of mortality in patients with osteosarcopenia versus controls, summarizing the data derived from multivariate statistical analyses. In the case of univariate analyses, the number of confounders was posed equal to zero. Then, we calculated the risk ratios (RRs) with their 95% confidence intervals (CIs). Statistical significance was assessed using the random effects model and inverse-variance method [12].

Statistical heterogeneity of outcome measurements between different studies was assessed using the overlap of their confidence interval (95% CI) and expressed as I^2^. Data classification as having low heterogeneity was based on I^2^ from 30 to 49%, moderate heterogeneity from 50 to 74%, and high heterogeneity from 75% and above [13]. In case of high heterogeneity, a random-effect meta-regression was planned to explore potential sources of variability that could affect estimate rates among studies [14]. We plan to consider as moderators mean age of the population, percentage of females, number of adjustments in multivariate analyses (in univariate analyses was posed equal to zero), and follow-up in years, but the main outcome did not suffer on any statistical heterogeneity.

Publication bias was assessed by visually inspecting funnel plots and using the Egger bias test [15]. In case of statistically significant publication bias, the trim-and-fill analysis was used [15]. For all analyses, a P-value less than 0.05 was considered statistically significant. All analyses were performed using STATA version 14.0 (StataCorp).

## Results

### Literature search

Among the 231 studies initially identified, we screened 114 records and retrieved 13 full texts. At this level, two studies were excluded: one was a review [7, 16], one did not report meta-analyzable data on mortality (only included in a composite outcome) [17], and one had limited data about the diagnosis of osteosarcopenia [18]. Finally, we included nine cohort studies [19–27]. The literature search selection is summarized in the PRISMA flowchart (**Supplementary Fig. 1)**.

### Descriptive characteristics

Table 1 shows the main descriptive characteristics of the studies included. Overall, the nine cohort studies included a total of 14,429 participants, followed up for a mean of 6.6 years. They aged a mean of 70 (SD = 6) years, and they were prevalently females (64.5%). The studies were conducted on all continents except for Africa, mainly Asia (*n* = 4), Europe (*n* = 2), South America (*n* = 2), and Oceania (*n* = 1). Among the main conditions considered, three studies were conducted among community-dwelling older people, while the other six considered specific medical conditions, such as cirrhosis, hip fracture, or similar (see Table 1 for further details). Regarding the diagnosis of sarcopenia, five studies used the criteria proposed by international societies that associated the evaluation of body composition parameters with muscle strength and/or physical performance, one study used phase angle parameters, and the other three studies, criteria specific for the population examined; similarly, the diagnosis of osteoporosis was made in six studies using a T-score less than − 2.5 SD, while two studies used less than one SD, and one study, criteria specific for the population included (Table 1).

**Table 1:** Descriptive characteristics of the studies included

Author, year	Country	Number of people with osteosarcopenia	Sample size total	Mean age (total sample)	SD age (total sample)	% of females (total sample)	Main condition	Follow-up (years)	Criteria to define sarcopenia	Criteria to define osteoporosis	Number of adjustments	List of adjustments
Balogun, 2019	Australia	86	1032	69.2	7.4	52	community-dwelling older adults	10	being in the lowest 20% of the sex-specific distribution of muscle mass and strength respectively	T-scores of the total hip and/or lumbar spine of less than − 1	4	age, sex, physical activity, 25-hydroxyvitamin-D
Kara, 2023	Turkey	40	104	74.7	8.7	71.1	recovery after vertebroplasty	5	A Psoas Muscle Index (PMI) < 540 mm2 /mm2 for male patients and < 360 mm2 /m2 for female patients, based on a previous study of a Turkish population	Based on a previous study of a Turkish population, osteoporosis was defined as an Hounsfield Unit value < 102 on Tomography	0	None
Paulin, 2023	Sweden	29	1044	75.2	0.1	not reported	older adults (75 years old)	10	EWGSOP (European Working Group on Sarcopenia in Older People) revised edition (2019)	T-score ≤ − 2.5 SD	5	smoking, alcohol, polypharmacy, albumin, and CRP (C reactive protein)
Saeki, 2023	Japan	24	126	70.5	not reported	38.9	cirrhosis	3	revised criteria of the Japan Society of Hepatology (second edition)	T-score ≤ − 2.5	1	Child-Pugh
Salech, 2020	Chile	183	2372	72	6.4	68.5	community-dwelling older adults	12	EWGSOP1 validated for Chilean population	T-scores < − 2.5	6	age, gender, comorbidity, smoking, polypharmacy, and mobility
Sepulveda Loyola, 2023	Chile		323	68	6	77	community-dwelling older adults	9	phase angle	T score <-1	0	None
Shimada, 2023	Japan	669	8895	73.5	5.4	51.7	older adults	5	EWGSOP2 with cuf-offs values recommended by AWGS (Asia Working Group for Sarcopenia)	T-scores < 2.5	12	age, sex, hypertension, heart disease, pulmonary disease, diabetes, osteoarthritis of the knee, body mass index (BMI), walking speed, physical activity, MMSE and (GDS-15)
Xiang, 2023	China	23	209	58.4	15.3	52.6	hemodialysis	4	AWGS criteria	T-scores < 2.5	6	age, sex, dialysis vintage, diabetes, cardiovascular disease, and fracture history
Yoo, 2018	Korea	93	324	77.8	9.7	75.9	hip fracture	1	AWGS criteria	T-scores < 2.5	4	age, gender, BMI, Koval
**Total**		**1147**	**14,429**	**70**	**6**	**64.5**		**6.6**			**4**	

### Osteosarcopenia as a risk factor for mortality: meta-analysis

**Fig. 1:**
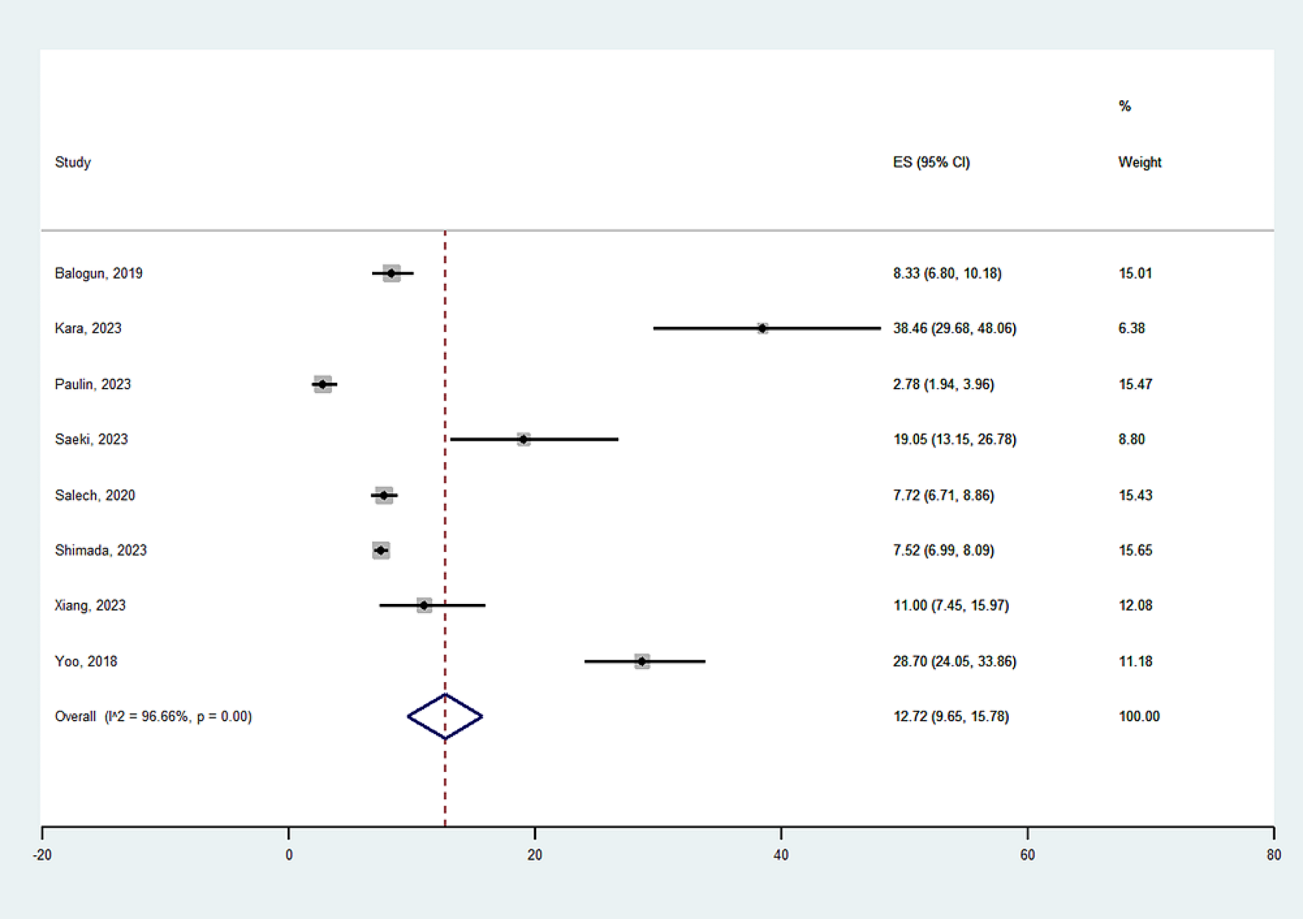
Prevalence of osteosarcopenia in the studies included

Figure 1 shows the prevalence of osteosarcopenia in the studies included. Overall, the studies reported that 1,147 over 14,429 participants suffered from osteosarcopenia for a weighted prevalence of 12.72% (95%CI: 9.65–15.78) (Fig. 1). The prevalence largely varied from 2.78% [21] to 38.46% [20], leading to a substantial heterogeneity (I^2^ = 99%).

**Fig. 2:**
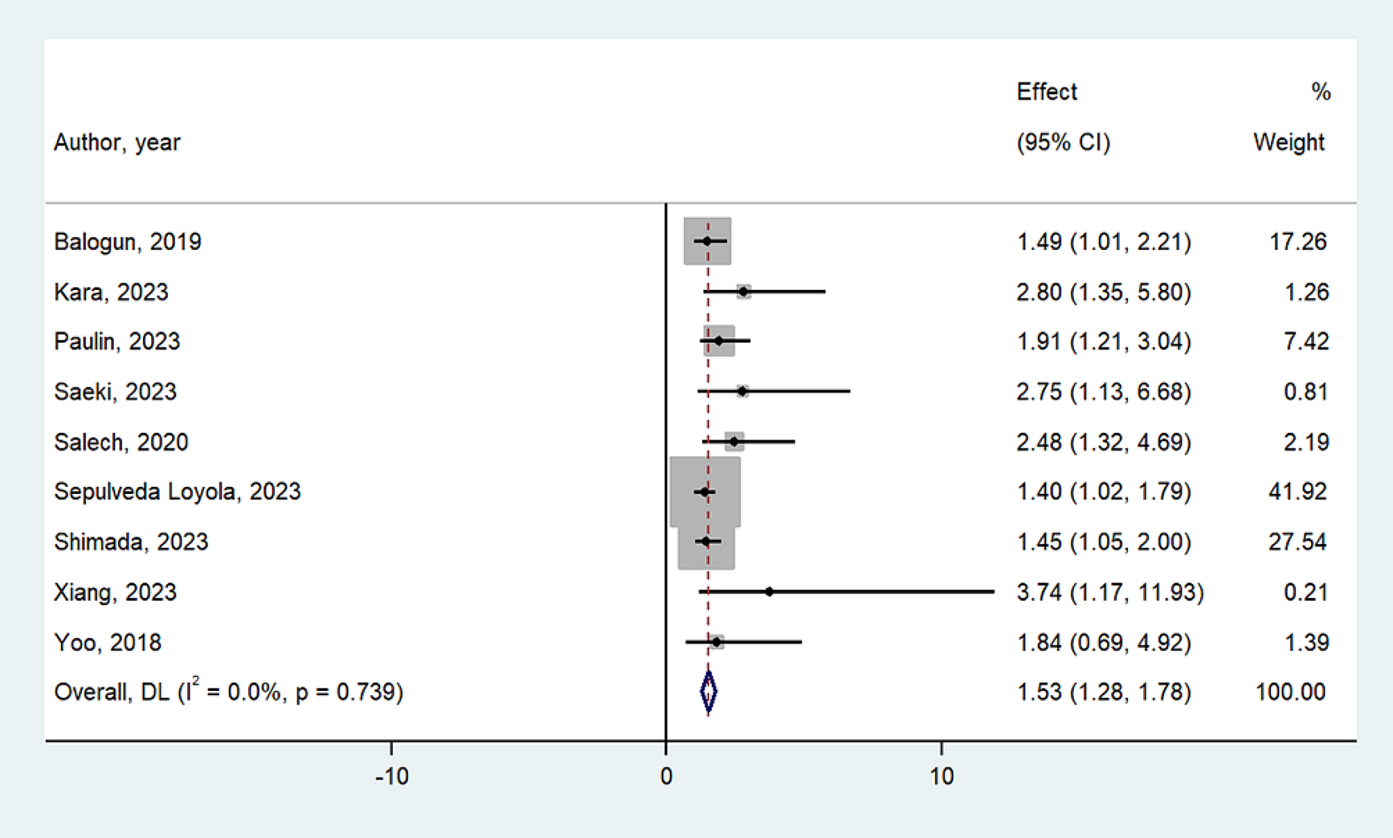
Meta-analysis of osteosarcopenia as predictor factor for mortality

Figure 2 shows the association between osteosarcopenia at the baseline and mortality. After adjusting the analyses for a mean of four potential confounders (see the list in Table 1), the presence of osteosarcopenia significantly increased the risk of mortality in the cohort studies included by 53% (RR = 1.53; 95%CI: 1.28–1.78). This analysis was not affected by any significant heterogeneity (I^2^ = 0%), and all the studies reported a significant association between osteosarcopenia and mortality except for one [26]. 

This outcome was, however, affected by the presence of publication bias (Egger’s test p-value < 0.0001): after using the trim-and-fill analysis, with four studies trimmed at the left of the mean, the association was only slightly reduced (RR = 1.48; 95%CI: 1.23–1.72).

### Risk of bias

The risk of bias evaluation is reported in **Supplementary Table 2**. Overall, the mean NOS was 8, with no study at possible high risk of bias. The main source of risk of bias was the short time of follow-up, less than 5 years.

## Discussion

In this systematic review with meta-analysis, including nine cohort studies with a total of 14,429 participants followed up for a mean of 6.6 years, we found that the presence of osteosarcopenia at the baseline increased the risk of mortality by 53%, also after accounting for several potential confounders. Even if the outcome suffers from publication bias, the trim-and-fill analysis only slightly attenuated our findings.

The first crucial epidemiological point is the high prevalence of osteosarcopenia found in our meta-analysis, i.e., about 12.7%. Osteosarcopenia represents a growing concern in aging populations. While individual prevalence estimates vary, studies suggest a substantial overlap between osteoporosis and sarcopenia, with prevalence rates ranging from 5 to 20% in older adults [28]. Of importance, the prevalence of osteosarcopenia is expected to rise in parallel with the aging population, placing a significant burden on healthcare systems and society [28]. Our review, using a meta-analytic approach confirms the epidemiological importance of this entity in geriatrics, across different clinical situations.

Overall, the pooled analysis indicated that osteosarcopenia significantly increased the risk of mortality, and the results were not affected by any heterogeneity, with practically all the studies reporting a significant positive association between osteosarcopenia and mortality. Our findings are in agreement with two previous reviews reporting that osteosarcopenia increased the risk of mortality [7, 16]. Even if these two systematic reviews increased the risk of our knowledge about this important topic, they could report only three [7] and five studies [16], respectively, therefore having more limited literature compared to our work. Indeed, according to several previous studies, both osteoporosis and sarcopenia individually increased the risk of mortality [5, 29]. Thus, the possibility that osteosarcopenia could significantly increase the risk of mortality is reasonable, as it involves the co-existence of the two aforementioned conditions [7]. Of importance is that the presence of osteosarcopenia significantly affects mortality rate independently from the definition used that was, however, of clinical heterogeneity for both, sarcopenia and osteoporosis. Altogether, our findings suggest that the importance of identifying osteosarcopenia does not stand in the diagnostic criteria used to identify it but in identifying this entity to effectively treat and prevent mortality.

Osteosarcopenia can increase the risk of mortality through different mechanisms. First, and most obviously, osteosarcopenia could increase the risk of fractures, including hip and falls [7, 30]. Both falls and fractures are widely known risk factors for mortality in older people [31]. In this regard, sarcopenia is a progressive and generalized skeletal muscle disorder characterized by the loss of muscle mass and function and is known to be associated with increased adverse outcomes related to fractures, falls, frailty, disability, and mortality [5]. Moreover, sarcopenia also represents a significant economic burden worldwide [32], with a remarkable prospected increase in the next 40 years [32]. At the same time, osteoporosis is a chronic skeletal disorder characterized by low bone mass and mineral density, along with the deterioration of bone–tissue microarchitecture, further leading to bone fragility and consequential susceptibility to fractures, disability, and mortality [29]. With the aging of the global population, these two conditions will become more prevalent, and the incidence of osteosarcopenia will thus increase dramatically in the upcoming decades [7]. Therefore, osteosarcopenia represents an important public health issue to which great attention should be paid globally, also because it significantly increases the risk of death independently from potential confounders.

The findings of this systematic review must be considered within its limitations. First, we could not estimate whether the risk of mortality caused by osteosarcopenia was higher compared to the presence of sarcopenia or osteoporosis alone due to insufficient original data. Second, even if the I^2^ was < 50%, the diagnostic criteria for osteosarcopenia may have affected the results from a clinical point of view, not leading to a univocal definition of this entity. For example, some studies included osteoporotic patients, but others involved osteopenic participants; similarly, sarcopenia was defined according to different criteria. Third, some studies explored osteosarcopenia among community dwellers, while others analyzed specific populations. Fourth, even if we used the results of multivariable analyses, the adjustment factors differed among studies.

In conclusion, our systematic review suggests that osteosarcopenia significantly increases the risk of mortality by about 53% compared to controls. Our results underline the need to consider the presence of osteoporosis in sarcopenic patients, and vice versa, since the combination of these two conditions, typical of older people, may lead to further adverse complications, such as mortality.

### Electronic supplementary material

Below is the link to the electronic supplementary material.

Supplementary Material 1

## Data Availability

Data are available upon request to the Corresponding Author, based on a reasonable request.
