# Supplementary material for: Osteosarcopenia increases the risk of mortality: a systematic review and meta-analysis of prospective observational studies
Source: Aging Clin Exp Res. 2024 Jun 18;36(1):132. doi: 10.1007/s40520-024-02785-9 (PMC11189340; doi:10.1007/s40520-024-02785-9)
Supplement: Supplementary file 1 — Supplementary Material 1 [file 40520_2024_2785_MOESM1_ESM.docx]

**Supplementary Figure 1. PRISMA flow-chart.**

PubMed (*n* = 65)
Web of Science (*n* = 80)
Embase (*n* = 86)
Total (*n* = 231)

Records removed *before screening*:

Duplicate records removed (*n* = 117)

**Identification**

Records screened

(*n* = 114)

Records marked as ineligible

(*n* =101)

Reports not retrieved

(*n* =0)

Reports sought for retrieval

(*n* =13)

**Screening**

Reports excluded:

Review (n=2)

No mortality data (n=1)

No Osteosarcopenia (n=1)

Reports assessed for eligibility (*n* =13)

Studies included in the systematic review & meta-analysis (*n* = 9)

**Included**

**Supplementary Table 1. Search strategies.**

| **Database** | **Search** | **#** |
| --- | --- | --- |
| **Pubmed** | (Osteosarcopenia OR Sarco-osteopenia OR Osteo-sarcopenia OR Osteosarcopenic) AND (mortality OR death) | **65** |
| **Web of Science** | osteosarcopenia (All Fields) and mortality OR death (All Fields) | **80** |
| **Embase** | 'osteosarcopenia'/exp AND ('mortality'/exp OR 'mortality' OR 'mortality model' OR 'death'/exp OR 'death' OR 'demise' OR 'lethal outcome' OR 'mors') | **86** |

**Supplementary Table 2. Evaluation of the risk of bias of the studies included.**

| ***NOS criteria*** | ***Balogun, 2022*** | ***Yoo, 2017*** | ***Kara, 2023*** | ***Sepulveda, 2023*** | ***Xiang, 2023*** | ***Shimada, 2023*** | ***Paulin, 2023*** | ***Salech, 2023*** | ***Saeki, 2023*** |
| --- | --- | --- | --- | --- | --- | --- | --- | --- | --- |
| A. Selection |  |  |  |  |  |  |  |  |  |
| 1. Representativeness of the exposed cohort | ★ | ★ | ★ | ★ | ★ | ★ | ★ | ★ | ★ |
| 2. Selection of the non-exposed cohort | ★ | ★ | ★ | ★ | ★ | ★ | ★ | ★ | ★ |
| 3. Ascertainment of exposure | ★ | ★ | ★ | ★ | ★ | ★ | ★ | ★ | ★ |
| 4. Demonstration that outcome of interest was not present at start of study | ★ | ★ | ★ | ★ | ★ | ★ | ★ | ★ | ★ |
| B. Comparability |  |  |  |  |  |  |  |  |  |
| 1. Comparability of cohort on the basis of the design or analysis | ★★ | ★★ | ★★ | ★★ | ★★ | ★★ | ★★ | ★★ | ★★ |
| C. Outcome |  |  |  |  |  |  |  |  |  |
| 1. Assessment of outcome | ★ | ★ |  | ★ |  |  | ★ | ★ |  |
| 2. Was follow-up long enough for outcomes to occur | ★ |  |  |  |  | ★ | ★ | ★ | ★ |
| 3. Adequacy of follow-up of cohorts | ★ | ★ | ★ | ★ | ★ | ★ | ★ | ★ |  |
| **Total (maximum of nine stars)** | **9** | **8** | **7** | **8** | **7** | **8** | **9** | **9** | **7** |
